# The Therapeutic Role of Monocyte Chemoattractant Protein-1 in a Renal Tissue Engineering Strategy for Diabetic Patients

**DOI:** 10.1371/journal.pone.0057635

**Published:** 2013-02-25

**Authors:** Hao Yin, Ming Gao, Lara Leoni, Huifang Han, Xing Zhang, Zhiren Fu

**Affiliations:** 1 Department of Surgery, Shanghai Changzheng Hospital, Shanghai Second Military Medical University, Shanghai, China; 2 Department of Surgery, The University of Chicago, Chicago, Illinois, United States of America; 3 Integrated Small Animal Imaging Research Center, The University of Chicago, Chicago, Illinois, United States of America; 4 Department of Surgery, Shanghai Chongming Hospital, Chongming County, Shanghai, China; 5 School of Engineering and Applied Sciences, Harvard University, Cambridge, Massachusetts, United States of America; Universidade de Sao Paulo, Brazil

## Abstract

In this study we aim to boost the functional output of the intra-kidney islet transplantation for diabetic patients using a tissue engineered polymeric scaffold. This highly porous electrospun scaffold featured randomly distributed fibers composed of polycaprolactone (PCL) and poliglecaprone (PGC). It successfully sustained murine islets *in vitro* for up to 4 weeks without detected cytotoxicity. The *in vivo* study showed that the islet population proliferated by 89% within 12 weeks when they were delivered by the scaffold but only 18% if freely injected. Correspondingly, the islet population delivered by the scaffold unleashed a greater capability to produce insulin that in turn further drove down the blood glucose within 12 weeks after the surgery. Islets delivered by the scaffold most effectively prevented diabetic deterioration of kidney as evidenced by the lack of a kidney or glomerular enlargement and physiological levels of creatinine, urea nitrogen and albumin through week 12 after the surgery. Unlike traditional wisdom in diabetic research, the mechanistic study suggested that monocytes chemoattractant protein-1 (MCP-1) was responsible for the improved preservation of renal functions. This study revealed a therapeutic role of MCP-1 in rescuing kidneys in diabetic patients, which can be integrated into a tissue engineered scaffold to simultaneously preserved renal functions and islet transplantation efficacy. Also, this study affords a simple yet effective solution to improve the clinical output of islet transplantation.

## Introduction

According to the American Diabetes Association, the diabetes inflicts 25.8 million patients in the U.S in 2011 and will dramatically increase the likelihood of other diseases, such as heart diseases, kidney failure, nervous system diseases, etc. Particularly, type I diabetes resulting from the autoimmune destruction of functional pancreatic beta-cells responsible for producing insulin heavily burdens the children as well as adult patients. To restore the depressed or lost insulin production, scientists have extensively explored islet transplantation as a therapeutic solution in the past few decades but have met limited clinical success [1]. A number of challenges have thwarted this endeavor, including inflammation, lack of vasculature, etc, which account for the rapid loss of functional islet population after transplantation [2], [3]. In addition, recent tissue engineering research has revealed the *in vivo* tissue regeneration and/or remodeling is heavily regulated by the immune system that is typically activated by the introduction of foreign materials and traumatic surgery [4].

In this study, we pioneered the employment of an electrospun composite scaffold of polycaprolactone (PCL) and poliglecaprone (PGC) as the delivery vehicle for syngeneic murine islet transplantation to improve the clinical performance of islet transplantation in diabetic patients. PGC and PCL are both FDA approved degradable suture materials and thus the composite scaffold is expected to provide a temporal structural support for the islet population to integrate with the host. Our investigation showed that the scaffold increased the proliferation of transplanted islets and their ability to regulate blood glucose and glomerular functions in diabetic mice compared to those that received freely injected islets. Furthermore, a mechanistic study revealed that monocytes chemoattractant protein-1 (MCP-1) was responsible for this improvement, suggesting a promising therapeutic candidate in future renal tissue engineering strategy for diabetes.

## Materials and Methods

### The Fabrication and the Morphological Characterization of the Electrospun Scaffold

PGC (Advanced Inventory Management, Mokena, IL) and PCL (Absorbable Polymers, Birmingham, AL) were dissolved (weight ratio of 1∶3) in 1,1,1,3,3,3-Hexafluoro-2-propanol (HFP) (Sigma Aldrich, St. Louis, MO) to achieve a total concentration of 12% (w/v). The solution was then loaded into a syringe capped with a 27 gauge blunt needle and distanced at 25 cm from the collection board. 0.5 mL of the solution was electrospun to the collection board at a voltage of 30 kV and a feeding rate of 3 ml/hr. Thereafter, the scaffold was retrieved from the board and desiccated in vacuum for 24 hr prior to subsequent analyses. A square specimen measuring 1 cm×1 cm was cut from the scaffold and sputter-coated by gold. A scanning electron microscope (SEM) (Philips SEM 510) was employed to take images of the specimen at an acceleration voltage of 30 kV.

### MIP-luc Transgenic Mice and the Creation of Diabetic Mice

MIP-luc transgenic mice (in a C57BL/6 background) were generated, where the transgene comprises the MIP promoter fragment driving the expression of the firefly luciferase (MIP-luc) [5], [6]. Beta cells from MIP-luc mice can be visualized using bioluminescent imaging and their mass is correlated with the bioluminescent signal [7]. Hemizygous MIP-luc transgenic mice (littermates from a single homozygous male MIP-luc mouse) were treated with a single intraperitoneal (IP) injection of Streptozotocin (STZ) (150 mg/kg, Sigma Chemical, St. Louis, MO) to induce diabetes. Diabetic mice with non-fasted blood glucose values >400 mg/dl for more than 2 consecutive days (SureStep; Lifescan, Milpitas, CA) were considered diabetic. The animal protocol of this study was approved by the Animal Care and Use committee of the Second Military Medical University and Shanghai Changzheng Hospital (Permit Number. 08–0086).

### In vitro Biocompatibility Analysis and Measurement of Insulin

Syngeneic islets from MIP-luc transgenic C57BL/6 mice were isolated following intraductal collagenase digestion (Collagenase P, 0.3 mg/ml; Roche, Indianapolis, IN) and purification by Ficoll gradient centrifugation (Sigma Aldrich, St. Louis, MO) as previously described [8], [9]. Circular specimens (D = 6.5 mm) were cut from desiccated scaffold and plated into a 96-well tissue culture plate (TCP). All specimens were incubated with 70% ethanol for at least 15 min and thoroughly rinsed with sterile phosphate buffer saline (PBS). Freshly collected islets were seeded (100 islet equivalents/scaffold) on the scaffold or directly on the bottom surface of the plate in islet growth media, and incubated for up to 4 weeks at 37°C with 5% CO_2_. The viability of the islet population was measured by the tetrazolium compound [3-(4,5-dimethylthiazol-2-yl)-5-(3-carboxymethoxyphenyl)-2-(4-sulfophenyl)-2H-tetrazolium, inner salt] (MTS) (Promega Corporation, Madison, WI) weekly per the manufacturer’s protocol. For the *in vitro* insulin secretion assay, freshly harvested islets were seeded on the scaffold (100 islet equivalents/scaffold) contained in a 96-well TCP and cultured for 24 hr at 37°C with 5% CO_2_. The supernatant of the cell media was collected at 3 hr and 24 hr, and measured by an RIA kit (Linco Research, Inc., Si. Charles, MO) per manufacturer’s protocol. In the control group, 100 islet equivalents were directly cultured in the 96-well TCP.

### Transplantation of ISLETS into Diabetic Mice

Islets were harvested and scaffold samples (1 mm×1 mm) fabricated and sterilized as described above. Islets were seeded (100 islet equivalents/scaffold) on the scaffold in minimal volume of islet growth media and incubated for 3 hr at 37°C with 5% CO_2_ prior to the surgical implantation. Recipient mice were anesthetized by vaporized 2.5% isoflurane via inhalation. The islets with or without a scaffold were surgically placed under the kidney capsule. Each mouse received one set of islet/scaffold or freely injected islets in one kidney. The experiment comprised four groups with 5 mice in each group: (1) sham group which received neither islets nor scaffolds; (2) scaffold group which received scaffolds only; (3) islet group which received freely injected islets; and (4) islet/scaffold group which received islets on the scaffold. All mice were sacrificed 12 weeks after the surgery.

### In vivo Proliferation and Functions of Transplanted Islets

Bioluminescent optical imaging was performed using a Xenogen IVIS 200 imaging system (Xenogen, Alameda, CA) as previously described [6]. Briefly, MIP-luc mice were fasted for 4 h, shaved, and anesthetized with vaporized isofluorane using the Xenogen system. Mice were placed on their sides on the imaging stage and an overlay image was initially taken. Then mice were injected i.p. with 15 mg/ml D-luciferin in sterile PBS (150 mg/kg) and exactly 14 min after the injection, a bioluminescent image was captured with an exposure time of 1 minute. Subsequent image processing, including quantification of bioluminescence, was conducted using the Living Image Software v. 2.05 (Xenogen).

Blood samples were drawn from the tail vein of each mouse immediately before the surgery and then every 4 weeks post-surgery. All mice were non-fasted before the blood collection. Blood glucose was measured by a OneTouch Ultra glucometer (Lifescan, Johnson & Johnson, Milpitas, CA). Serum insulin was assayed with rat insulin ELISA kit with mouse insulin standards (Crystal Chem Inc., Chicago, IL) and serum C-peptide 2 assayed with a rat/mouse C-peptide 2 ELISA kit (Millipore, Bellirica, MA, USA) per manufacturers’ protocols.

An oral glucose tolerance test (OGTT) was performed in mice 4 weeks after the surgery. Briefly, mice were fasted for 16 hr and blood samples were collected as described above to obtain the baseline glucose level (0 min). Thereafter, each mouse received 2 g/kg body weight of a 100 mg/ml glucose solution (Sigma Aldrich) in sterile water delivered by oral gavage. At 30, 60 and 120 min after the glucose administration, blood samples were collected to measure the glucose level as described above.

To confirm the effect of transplanted islets, 4 weeks after the surgery the kidney housing the islet and/or scaffold was surgically removed while the mice were anesthetized by vaporized isoflurane. The blood glucose, insulin and C-peptide 2 concentrations were measured 2 weeks after the kidney removal as described above.

### Histological Evaluation of Retrieved Islets and Kidneys

After euthanasia, harvested kidneys were weighed on a top-loading digital scale. Then kidneys and islets was embedded in Tissue-Tek OCT (Sakura Finetek, Torrance, CA), and snap-frozen in liquid nitrogen. Samples were sliced into 4 µm thick specimens that were serially collected on charged glass slides. The histology of samples was studied by staining with hematoxylin and eosin (H&E) (Sigma Aldrich). Insulin-secreting cells were stained with anti-insulin antibody (Abcam, Cambridge, MA, USA) and counterstained with hematoxylin. One slide from each mouse that showed at least 5 glomeruli was selected for the calculation of glomerular area. All mice from each group were included, thus giving at least 25 data points for each group in the glomerular area calculation. The area of glomeruli (the structure contained in the Bowman’s capsule) was calculated in each harvested kidney tissue from every treatment group. All slides were scanned by a CRi Pannoramic Scan Whole Slide Scanner and images were processed using the Pannoramic Viewer (3D Histech, Budapest, Hungary), which allows a continuous selection of magnification.

### Evaluation of Renal Functions after the Isle Transplantation

Blood and urine samples from each mouse were collected immediately before the surgery and then every 4 weeks after the surgery. Plasma cytokines of interest, including MCP-1, interlukin-6 (IL-6) and interferon gamma (IFNγ), were analyzed by the Immunoassays & Multiplex Kits (EMD Millipore, Billerica, MA) per the manufacturer’s protocol. Kidney proteins, including blood creatinine (Abcam), blood urea nitrogen (Bio Scientific Corp, Austin, TX), urine creatinine (Abcam), urine albumin (Abnova, Walnut, CA), were assayed following manufacturers’ protocols.

### The Mechanistic Study of MCP-1 on Retaining Renal Functions in Diabetic Mice

STZ was given at a dose of 50 mg/kg every two days for a total of 5 doses to induce diabetes and to allow a gradual renal deterioration before the administration of insulin and MCP-1. Thereafter, each mouse received one LinBit insulin tablet (LinShin Canada Inc, Toronto, Ontario) via subcutaneous implantation every 4 weeks. In addition, one group of insulin-treated mice (n = 5) received recombinant mouse MCP-1 (BD Pharmingen, San Jose, CA) dissolved in sterile phosphate buffered saline (PBS) by intraperitoneal injection every week until euthanasia (0.5 mL of 20 µg/mL). Kidney proteins were assayed every 4 weeks as described above. Mice were euthanized 12 weeks after the insulin administration and kidney tissues were analyzed as described above.

### Statistical Analysis

All images were processed by ImageJ. All data was analyzed using student t-test or ANOVA with a Tukey test where applicable. The significance level was set at 95% (a = 0.05). All results were presented as mean±standard deviation (SD).

## Results

### The Electrospun Scaffold could Sustain the Islet Population in vitro

**Figure 1:**
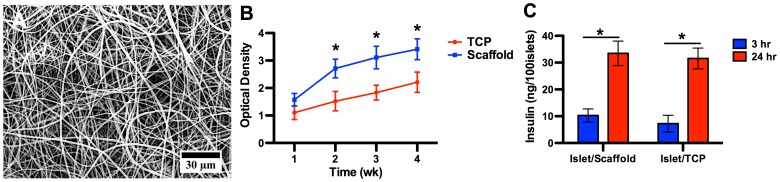
The in vitro characterizations of the electrospun scaffold. (A) The SEM micrograph of the electrospun scaffold. The scaffold comprised randomly distributed micro-fibers with a highly porous micro-structure, analogous to native extracellular matrix. (B) Proliferation assay of islets seeded on the scaffold and tissue culture plate (TCP). Islets cultured on the scaffold saw a steady increase through week 4 and outgrew its peer on the TCP as early as on week 2. This proliferative advantage was sustained through week 4, evidencing that the scaffold provided a more favorable biophysical environment than standard tissue culture surface for islet growth. A star indicates a statistical difference between scaffold and TCP groups on respective time points (n = 5). (C) *In vitro* insulin secretion by islets on the scaffold and TCP at 3 hr and 24 hr after seeding. A difference was observed between 3 hr and 24 hr within each substrate group but no difference between groups at either time points. The comparable insulin concentrations between the islet/scaffold and islet/TCP groups at both time points confirmed that the scaffold could equally recoup the critical insulin secretion capability within 24 hr.

The SEM micrograph showed that the scaffold featured randomly distributed fibers on the micro and nano-scale (Figure 1A). The majority of fibers had a diameter between 800 nm to 1200 nm, physically analogous to those protein fibers and bundles in the native extracellular matrix. It should be noted that each individual fiber was a composite of PGC and PCL.

To assess the biocompatibility of electrospun PGC/PCL scaffold, we measured the proliferation of harvested mouse islets cultured on the scaffold for up to 4 weeks using the MTS assay (Figure 1B). The islet population witnessed a steady growth within 4 weeks on the scaffold without detected cytotoxic effects from the scaffolding materials. Also, starting from week 2, the islet population on the scaffold outgrew its peer on the TCP, suggesting that the electrospun scaffold provided a more favorable biochemical and biophysical environment for islets to adhere and grow, and that these scaffolding materials, either intact or degraded, possessed no cytotoxicity to islets. The scaffold allowed a rapid and convenient delivery of islets in surgical procedures (Figure S1 in the supplement).

A readily secretion of insulin by transplanted islet governs the ultimate success of this therapeutic strategy. The concentrations of insulin secreted by islets cultured on the scaffold were 10.27±2.45 ng/100 islets at 3 hr and 33.45±4.56 ng/100 islets at 24 hr, respectively. Correspondingly, islets on the TCP yielded insulin at 7.24±3.11 ng/100 islets at 3 hr and 31.54±3.89 ng/100 islets at 24 hr, respectively (Figure 1C). Within both groups, a significant increase was observed between 3 hr and 24 hr, showing that the islets readily secreted insulin on both substrates. However, no difference was observed between the two groups at either time points, suggesting that the scaffold was an equally favorable substrate for islets to attach and to immediately secrete insulin. This result warrants the *in vivo* application of this scaffold as a delivery vehicle for islets transplantation in diabetic mice.

### Islets Delivered by the Scaffold Witnessed the Greatest Proliferation and Functional Output

**Figure 2:**
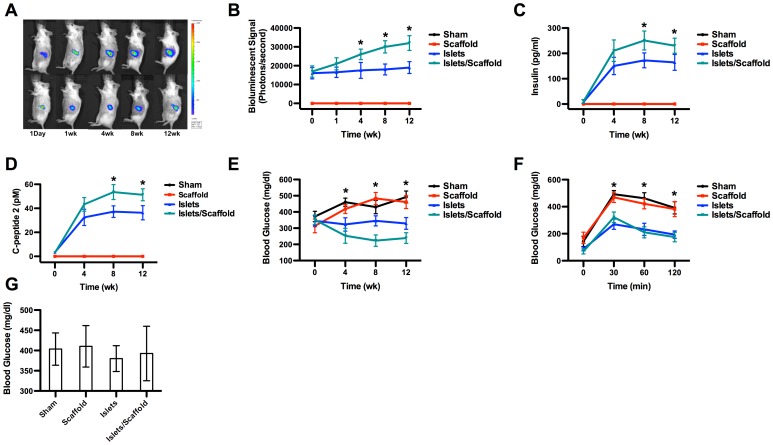
The in vivo proliferative and functional evaluations of transplanted islets. (A) Representative bioluminescent images post-transplantation. Mice from the islet/scaffold group were shown in the top row while those from the islet group in the bottom row. Mice in the sham and scaffold groups were not shown in the heat map since no islet transplantation was carried out. (B) *in vivo* proliferation of transplantation islets. The computational quantification of bioluminescent signal showed that the islet population in the islet/scaffold group started to outnumber its peers from the islet group on week 4 and sustained this advantage through week 12. The growing difference between mice from the islet/scaffold and islet groups within this 12-week time window suggests that the scaffold unleashed a greater capability to promote the islet proliferation. (C) Insulin production by transplanted islets. (D) C-peptide 2 production by transplanted islets. Mice from both the islet/scaffold and islet groups witnessed a large increase of insulin and C-peptide 2 within the first 4 weeks. However, on week 8 and week 12, islets in the islet/scaffold group outperformed those in the islet group. (E) Blood glucose production. By week 4, mice in the islet and islet/scaffold groups witnessed a significant decrease of blood glucose compared to their counterparts in the sham and scaffold groups. However, no difference was observed between the islet and islet/scaffold groups. On week 8 and 12, the blood glucose in mice from the islet/scaffold group was significantly lower than those from the islet group with mice from the sham and scaffold groups suffering from hyperglycemia. (F) Oral glucose tolerance test (OGTT). Following the oral administration of glucose, mice from the islet and islet/scaffold groups saw a temporary increase of glucose level within the first 30 min but the glucose level rapidly fell down to physiological levels by 120 min. On the contrary, mice from the sham and scaffold groups suffered a consistent high glucose level despite a minor decrease by 120 min. (G) Glucose levels after the removal of transplanted islets. Two weeks after the removal of transplanted islets, mice from the islet and islet/scaffold group yielded a comparably high blood glucose level to those in the sham and scaffold groups. A star indicates a statistical difference among groups at respective time points (n = 5). The black line (sham group) was masked by the red line (scaffold group) in panel B, C and D because the readout from these two groups were nearly identical.

After the surgery, those mice that received transplanted islets were non-invasively monitored by bioluminescent imaging for 12 weeks (Figure 2A and 2B). The heat map of the transplantation site revealed that islet populations in both the islet group and islet/scaffold group experienced a sustained increase. However, the islets in the islet/scaffold group consistently outnumbered their freely injected counterparts from week 4 post-surgery. Within a 12-week period, the bioluminescent signal witnessed an increase of 89% in the islet/scaffold group but only 18% in the islet group, further attesting the beneficial effect conferred by the scaffold.

We also measured three critical factors that determined the success of this therapeutic strategy, the serum insulin, C-peptide 2 and blood glucose (Figure 2C–2E). On week 4 the insulin concentration in the islet/scaffold group reached 210.54±42.33 pg/ml and 150.27±34.11 pg/ml in the islet group without a significant difference between the two groups. Thereafter, the insulin concentration in the islet/scaffold group reached 250.92±37.34 pg/ml on week 8 and 230.21±29.65 pg/ml on week 12, respectively. In contrast, the insulin concentration in the islet group reached 172.34±29.38 pg/ml on week 8 and 164.53±31.23 pg/ml on week 12, respectively. On both week 8 and week 12, a significant difference was observed between these two groups, suggesting that the scaffold promoted islet proliferation and functional output *in vivo* on a long term basis. The fact that no insulin was detected in the scaffold or sham groups suggested that the detected insulin in the islet and islet/scaffold groups were secreted by transplanted islets, rather than endogenous ones. Correspondingly, the profile of C-peptide 2 secretion paralleled that of insulin. No difference was observed on week 4 between the islet (32.45±6.77 pM) and the islet/scaffold groups (43.27±5.67 pM). However, a significant difference was observed on week 8 (37.21±4.82 pM in the islet group and 53.52±6.21 pM in the islet/scaffold group) and week 12 (36.32±5.91 pM in the islet group and 51.23±4.98 pM in the islet/scaffold group). No C-peptide 2 was detected in either the sham or scaffold group at any time points within 12 weeks. The blood glucose level of the islet (323±41 mg/dl) and islet/scaffold (253±46 mg/dl) group was significantly lower than those of the sham (460±25 mg/dl) and the scaffold (418±28 mg/dl) groups on week 4. Furthermore, on week 8 and 12 the glucose level of the islet/scaffold group was significantly lower than the other three groups. On week 8, the glucose concentrations were 430±41 mg/dl in the sham group, 483±37 mg/dl in the scaffold group, 345±31 mg/dl in the islet group and 223±35 mg/dl in the islet/scaffold group. On week 12, the glucose concentrations were 492±37 mg/dl in the sham group, 461±40 mg/dl in the scaffold group, 329±36 mg/dl in the islet group and 239±33 mg/dl in the islet/scaffold group. The OGTT result further confirmed that transplanted islets effectively capped the glucose spike at 30 min after the oral administration and returned the glucose level to the physiological range by 120 min (Figure 2F). Mice without islet transplantation suffered from a significantly higher glucose spike at 30 min and a sustained high glucose level through 120 min. The result that the blood glucose of islet and islet/scaffold groups reached a comparable level to those of the sham and scaffold groups after 2 weeks after the transplanted islets were removed spoke to the fact that transplanted islets were the effective regulator of blood glucose in these mice (Figure 2G). Meanwhile, the insulin and C-peptide concentrations were below the detection level.

**Figure 3:**
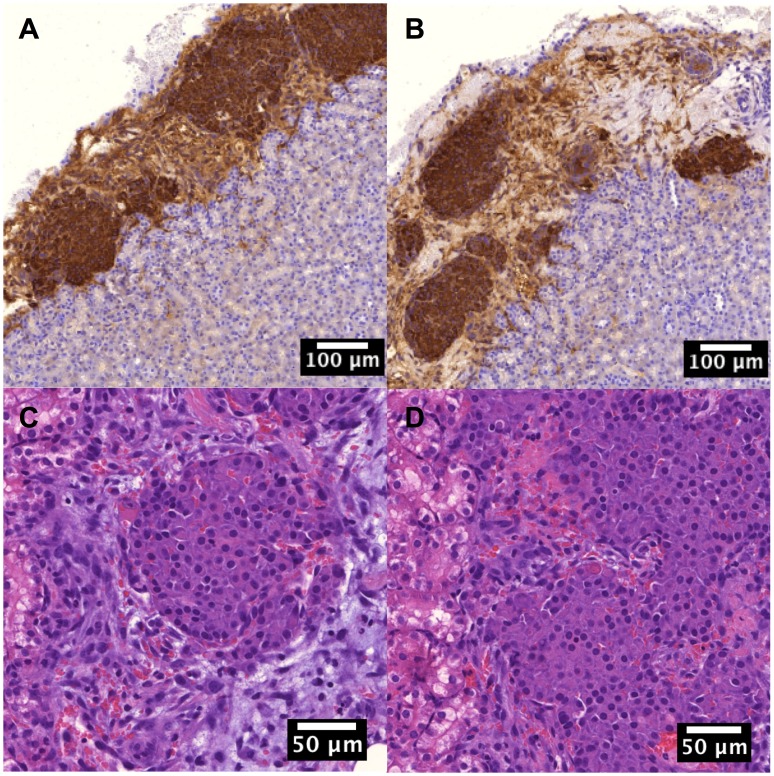
The morphological characterizations of transplanted islets. (A) Insulin-secreting cells from the islet/scaffold group. (B) Insulin-secreting cells from the islet group. The immunohistochemistry staining showed that the islet/scaffold group possessed more insulin-secreting cells, suggesting the proliferative benefit from the scaffold. (C) Histology of islets from the islet/scaffold group. (D) Histology of islets from the islet group. No density difference of inflammatory cells was evident, suggesting that scaffolding materials did not solicit undesired immune responses.

The immunohistochemistry staining confirmed that transplanted islets in both the islet and islet/scaffold groups readily secreted insulin (Figure 3A and 3B) through week 12. Moreover, the histology of islet transplants in both the islet and islet/scaffold groups showed comparable level of inflammatory cell invasion (Figure 3C and 3D), suggesting that the employment of scaffold did not lead to an increased local inflammation.

### Islets Delivered with Scaffolds Preserved Renal Functions

**Figure 4:**
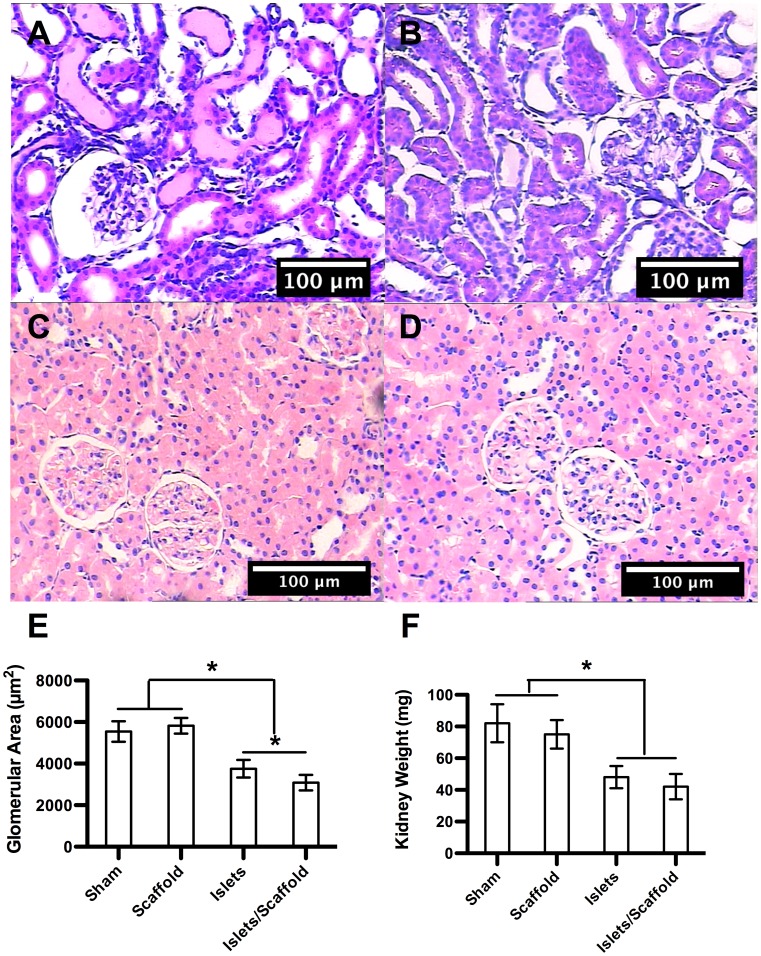
The evaluation of renal functions following the islet transplantation. The histology of renal tissues from sham (A), scaffold (B), islet (C) and islet/scaffold (D) groups. (E) Glomerular area (n≥25). (F) Kidney weight (n = 5). The histology showed significant edema in the kidney cortex in mice from the sham and scaffold groups, which resulted in an increase of glomerular area and overall kidney weight. On the contrary, transplanted islets in mice from the islet and islet/scaffold groups largely prevented the edema. The glomerular area in mice from the islet/scaffold group was smaller than those from the islet group, suggesting that the increased functional output of islets delivered by the scaffold better protected renal tissues. The significant glomerular enlargement in mice from the sham and scaffold groups could be attributed to compensate compromised renal functions, which is typically observed in diabetic patients. A star indicates a statistical difference between groups connected by a hanging bar.

The histological study demonstrated that the edema in the kidney cortex, a typical complication in diabetic patients, was significant in the sham and scaffold groups while largely absent in the islet and islet/scaffold groups (Figure 3A–3D). The glomerular areas from the sham, scaffold, islet and islet/scaffold groups were 5543±492 µm^2^, 5820±375 µm^2^, 3752±423 µm^2^ and 3084±372 µm^2^, respectively (Figure 3E). The islet/scaffold group had the smallest glomerular area followed by the islet group with the sham and scaffold groups featuring the largest ones. Similarly, the kidney weights from the sham, scaffold, islet and islet/scaffold groups were 82±12 mg, 75±9 mg, 48±7 mg and 42±8 mg, respectively (Figure 4F). The kidneys from the islet and islet/scaffold groups were significantly smaller than those from the sham and scaffold groups.

**Figure 5:**
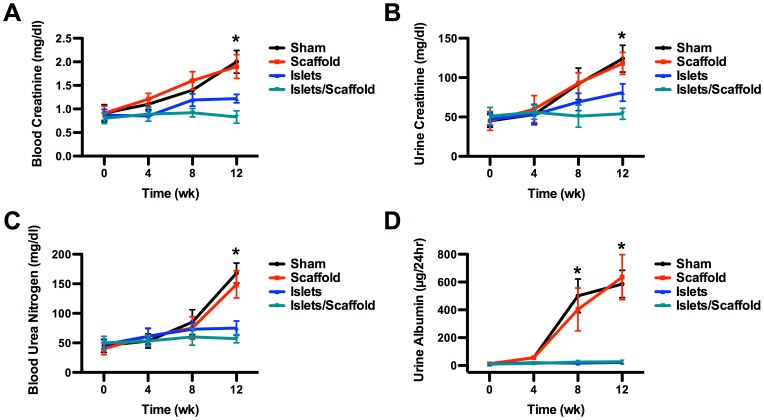
Plasma and urine protein concentrations following the islet transplantation. The concentrations of blood creatinine (A), urine creatinine (B), blood urea nitrogen (C) and urine albumin (D). All four proteins in mice from the sham and scaffold groups underwent a significant increase through week 12, suggesting the loss of renal functions. In contrast, transplanted islets in mice from the islet and islet/scaffold groups successfully contained these protein concentrations through week 12. Particularly, on week 12, the blood and urine creatinine concentrations were significantly lower in mice from the islet/scaffold group than those from the islet group, suggesting that the scaffold provided a long-term benefit for islet transplantation. A star indicates a statistical difference among groups at respective time points (n = 5 in all panels). The blue line (islet group) was masked by the teal line (islet/scaffold group) in panel D because the readouts were nearly identical.

Plasma and urine proteins in the sham and scaffold groups saw a sustained increase through week 12 after the surgery due to the deteriorating renal functions. In contrast, mice that underwent islet transplantation largely weathered through. By week 12 after the surgery, the blood creatinine concentration was 2.00±0.24 mg/dl in the sham group, 1.90±0.25 mg/dl in the scaffold group, 1.22±0.09 mg/dl in the islet group and 0.83±0.13 mg/dl in the islet/scaffold group (Figure 5A). Urine creatinine was 124.56±17.02 mg/dl in the sham group, 118.23±14.79 mg/dl in the scaffold group, 81.92±11.27 mg/dl in the islet group and 54.72±7.01 mg/dl in the islet/scaffold group, respectively (Figure 5B). A significant difference was observed in both blood and urine creatinine concentrations in mice between the islet and islet/scaffold groups. The blood urea nitrogen concentrations were 168.23±17.55 mg/dl in the sham group, 149.21±23.91 mg/dl in the scaffold group, 75.34±12.55 mg/dl in the islet group and 57.11±7.29 mg/dl in the islet/scaffold group, respectively (Figure 5C). The urine albumin concentrations were 586.21±98.11 µg/24 hr in the sham group, 635.19±22.45 µg/24 hr in the scaffold group, 22.59±7.41 µg/24 hr in the islet group and 27.91±5.51 µg/24 hr in the islet/scaffold group, respectively (Figure 5D). The concentrations of blood urea nitrogen and urine albumin in mice from the islet and islet/scaffold group were significantly lower than those from the sham and scaffold group but no difference was observed between the islet and islet/scaffold groups. The physiological levels of all these proteins were given in Table 1 in the supplement.

### MCP-1 was Responsible for the Improved Renal Functions in Mice from the Islet/Scaffold Group

**Figure 6:**
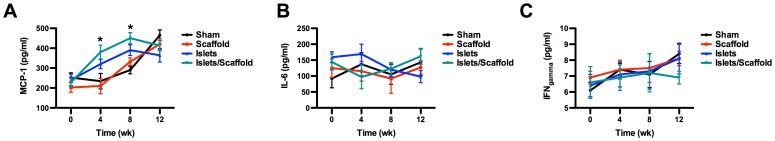
The concentration of immunological cytokines following the islet transplantation. Concentration of MCP-1 (A), IL-6 (B) and IFNγ (C). The MCP-1 concentrations in the sham and scaffold group steadily grew over 12 weeks, suggesting an increase of renal inflammation. In contrast, in the islet and islet/scaffold groups, the MCP-1 saw a temporary increase on week 4 and 8, which could be attributed to transplanted islets. A significant difference was observed between the islet and islet/scaffold groups on week 4 and 8. Thereafter, the MCP-1 concentration declined by week 12, which might be due to the down regulation from restored renal functions. No difference of IL-6 and IFNγ was observed among groups through week 12. A star denotes a statistical difference among groups at respective time points (n = 5 in all panels).

The activation of the immune system due to the transplantation surgery brings about a ripple effect across the entire *in vivo* system [10]. We hypothesized that immunological cytokines perturbed by the transplanted islets and/or scaffolding materials accounted for the improved renal functions. Therefore, we assayed prominent immunological cytokines involved in post-surgery tissue regeneration, including MCP-1, IL-6, and IFNγ (Figure 6). We discovered that the MCP-1 concentration in mice from the islet/scaffold group saw a rapid increase from 228.37±22.56 pg/ml to 380.21±35.09 pg/ml in the first 4 weeks after the surgery and outnumbered the other groups on week 4. This advantage was retained on week 8, thus exhibiting a drastically different profile of MCP-1 compared to those in the sham and scaffold groups. No difference of IL-6 or IFNγ among different treatment groups was observed through week 12. These findings led us to further investigate the mediating role of MCP-1 in retaining renal functions.

**Figure 7:**
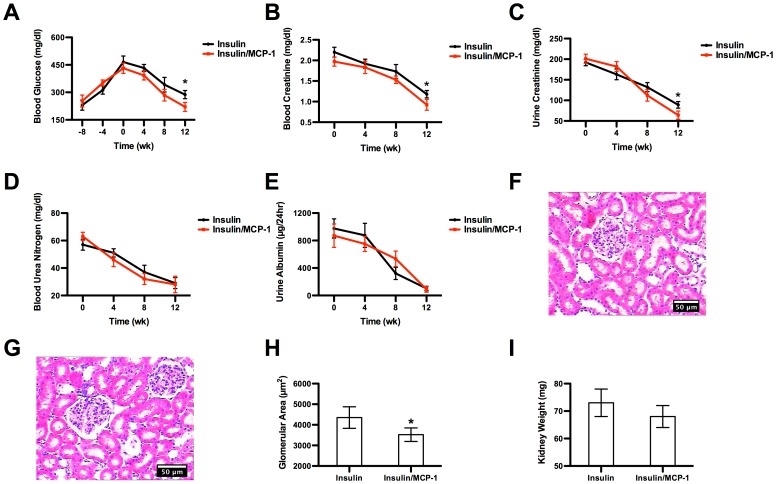
The mechanistic study of the role of MCP-1. (A) Blood glucose concentration before and after the insulin administration. After the insulin administration on week 0, the blood glucose witnessed a steadfast decline through week 12 in both groups. However, by week 12, the blood glucose level in the insulin/MCP-1 group was significantly lower than that in the insulin group. (B) Concentration of blood creatinine. (C) Concentration of urine creatinine. (D) Concentration of blood urea nitrogen. (E) Concentration of urine albumin. On week 12, the blood and urine creatinine concentrations in mice from the insulin/MCP-1 group were significantly lower than those from the insulin group, suggesting that MCP-1 played an active role in recouping renal functions. (F) Histology of glomeruli from the insulin group. (G) Histology of glomeruli from the insulin/MCP-1 group. (H) Glomerular area. (I) Kidney weight. The administration of MCP-1 led to the lack of glomerular enlargement, which was otherwise observed in mice in the insulin group.

The blood glucose level in diabetic mice was first allowed to exceed 400 mg/dl in an 8-week period to allow the compromise of renal functions while inducing diabetes. Thereafter, insulin was administered either with or without recombinant MCP-1 to recover those compromised renal functions. The administered insulin effectively drove down the blood glucose level to the physiological range by week 12 (Figure 7A). In the meantime, blood creatinine, urine creatinine, blood urea nitrogen and urine albumin all were reduced to near physiological levels, respectively (Figure 7B–7E). By week 12, the creatinine concentration in blood and urine in mice from the insulin/MCP-1 group were significantly lower than those from the insulin group. In addition, the glomerular area in mice from the insulin/MCP-1 group was significantly smaller than those from the insulin group but no difference was observed in the kidney weight between the two groups (Figure 7F–7I).

## Discussion

Tissue engineered polymeric scaffolds have been widely used to confer regenerational benefits to a great variety of native tissues, such as vasculatures, cardiac patch, bones, cartilage, etc [11]–[23]. Some tissue engineered scaffolds had led to remarkable clinical success [24]. Among the various fabrication methods, electrospinning has remained one of the most popular one and proved to be particularly useful in soft tissue regeneration. Its success in bone and cardiovascular tissue engineering has shed new lights on the therapeutic solution for diabetes. Autologous islet transplantation remains the most effective treatment for type I diabetes but suffers from a limited donor supply and poor survival rate after transplantation [1]–[3], [25]. To that end, we explored whether an electrospun scaffold could improve the survival and functional output of transplanted islets.

The highly porous microstructure of the electrospun scaffold is supposed to facilitate the islet adhesion, survival and proliferation for increased functional output like insulin secretion. PCL has been known to be the most durable material for electrospun scaffold with an *in vivo* degradation time around six months [26]. To keep pace with the production of native extracellular matrix, we incorporated PGC, which degrades much faster than PCL, into the scaffold to achieve an optimal degradation. Our *in vitro* results evidenced that the scaffolding materials possessed no cytotoxity for up to 4 weeks and that the physical microenvironment was more favorable for islets to adhere and grow than standard TCP surface. Moreover, islets cultured on the scaffold retained its critical physiological function by secreting insulin at a comparable level to their counterparts on the TCP on both 3 hr and 24 hr, suggesting that scaffolding materials did not compromise physiological functions of islets.

The most formidable challenge in tissue engineering research is to sustain implanted cells *in vivo* and to retain their normal functions for therapeutic purpose. To achieve this, it typically requires the rapid integration with the host, particularly the connection to the capillary network that is responsible for nutritional supply and removal of metabolic waste, followed by remodeling of *in vitro* engineered tissue to evolve into its native version [27], [28]. A proper engagement of the immune system is also demonstrated to be critical for the overall success of *in vivo* tissue regeneration [4]. To test the clinical potential of our novel strategy, we performed an extensive *in vivo* investigation to gauge the therapeutic effect of islets transplanted with scaffolds. The bioluminescent images and quantification showed that the scaffold greatly promoted the growth of islets compared to those freely injected within 12 weeks after the surgery as evidenced by the sustained increase of bioluminescent signal, which correlates with the mass of beta cells from MIP-luc mice mass [7]. By the end of week 12, the islet population had grown by 89% in the islet/scaffold group compared to just 18% in the islet group. These promising results were further strengthened by the quantitative measurements of serum insulin, C-peptide 2 and blood glucose concentration through week 12 post-surgery. On both week 8 and 12, the insulin and C-peptide 2 concentrations in the islet/scaffold group were consistently higher than those in the islet group. Correspondingly, the blood glucose in the islet/scaffold group was consistently lower than that in the islet group in the same time window. These results spoke to the fact that the scaffold promoted the functional output of transplanted islets. In addition, the OGTT result confirmed that transplanted islets were able to effectively prevent sudden glucose challenge. Moreover, two weeks after the transplanted islets were removed, the blood glucose in the islet and islet/scaffold groups increased to a comparable level to those in the sham and scaffold groups, evidencing that the transplanted islets were the effective regulator of blood glucose. This exciting phenomenon can be attributed to the fact that islets pre-seeded on the scaffold enjoyed a rapid growth after the transplantation, which translated into an increased functional output. In patients with advanced diabetes, the kidney would suffer from edema in the cortex with the glomerular basement membrane thickening, leading to an enlarged kidney. This is generally believed to compensate the reduced filtering capacity of kidney in diabetic patients. Our study demonstrated that the electrospun scaffold could prevent the pathological increase of glomeruli and kidney by retaining the function of transplanted islets, which pre-empted the hyperglycemia. The increased insulin secretion by islets delivered with the scaffold depressed the glucose level, which greatly alleviated the stress on renal tissues. And this achievement was documented by the most effective control of creatinine in the blood and urine in the islet/scaffold group. The results showing that mice from the islet/scaffold group outperformed the other three groups in regulating creatinine concentrations speak to the fact that the electrospun scaffold was a convenient way to boost the effectiveness of islet transplantation and thus contribute to the preservation of renal functions in diabetic patients.

Our discovery that MCP-1 spiked most in the islet/scaffold group on week 4 and 8, outnumbering its peers in the other three groups, led us to re-consider its role in diabetes. It is long believed that an increase of MCP-1 in diabetic patients is caused by renal inflammation that will gradually jeopardize the kidney [29]–[31]. In addition, previous study also showed that transplanted islets would increase MCP-1 concentration [10]. Interestingly, regenerative medicine research suggests that MCP-1 is up-regulated in tissue regeneration and would accumulate at injured vascular sites to initiate the cascade of immuno-responses that governs the ultimate tissue re-building [32]. Based on these findings, we tested whether the administration of MCP-1 could alleviate the pathological stress on renal tissues exerted due to diabetes. Our results showed that the administration of MCP-1 along with insulin into diabetic mice with moderate kidney failure restored the blood glucose, creatinine, urea nitrogen and urine albumin to physiological levels. Particularly, the creatinine concentrations in both blood and urine from the insulin/MCP-1 group were significantly lower than those from the insulin group, suggesting that the MCP-1 more potently recouped renal functions. Moreover, mice that received MCP-1 demonstrated a lesser degree of renal compromise as evidenced by the lack of glomerular enlargement. These results might be attributed to an MCP-1 mediated reno-vascular regeneration that accounted for the increased filtering capacity of glomeruli. The administration of MCP-1 together with insulin depressed the creatinine, blood urea nitrogen and urine albumin concentrations to a comparable level to those diabetic mice that underwent islet transplantation with scaffold. This suggests that a combination of the scaffold and MCP-1 might be able to boost the functional output of transplanted islets while preserve the renal functions.

### Conclusion

In this study we pioneered the employment of an electrospun scaffold to boost the functional output of transplanted autologous murine islets to treat type I diabetes. The *in vitro* results confirmed that it possessed no cytotoxity, could promote the islet proliferation and support the secretion of insulin for clinical applications. The *in vivo* results strongly evidenced that the scaffold promoted the growth of transplanted islets in STZ diabetic mice and restored insulin level in the blood, which effectively drove down the blood glucose concentration. As a result, the renal functions was maximally preserved. The mechanistic investigation held the increase of MCP-1 due to transplanted islets responsible for this improvement. These prominent results afford physicians a simple yet convenient alternative to traditional islet transplantation and shed new lights on the therapeutic use of MCP-1 to relieve kidney failure in diabetic patients. After confirming MCP-1 as a promising pharmaceutical candidate, we are now actively investigating how to engineer an MCP-1-eluting scaffold to simultaneously boost the function of transplanted islet and preserve renal functions.

## Supporting Information

Figure S1 **The surgical demonstration of the islet transplantation.** (A) Transplanted islets in the islet group. (B) Transplanted islets in the islet/scaffold group. The white arrow indicates the transplantation site.
(TIFF)

Table S1 **Concentrations of plasma and urine proteins in non-diabetic C57BL/6 mice (age and sex matched).**
(DOCX)
