# Supplementary figures and images for: The Therapeutic Role of Monocyte Chemoattractant Protein-1 in a Renal Tissue Engineering Strategy for Diabetic Patients
Source: PLoS One. 2013 Feb 25;8(2):e57635. doi: 10.1371/journal.pone.0057635 (PMC3581514; doi:10.1371/journal.pone.0057635)

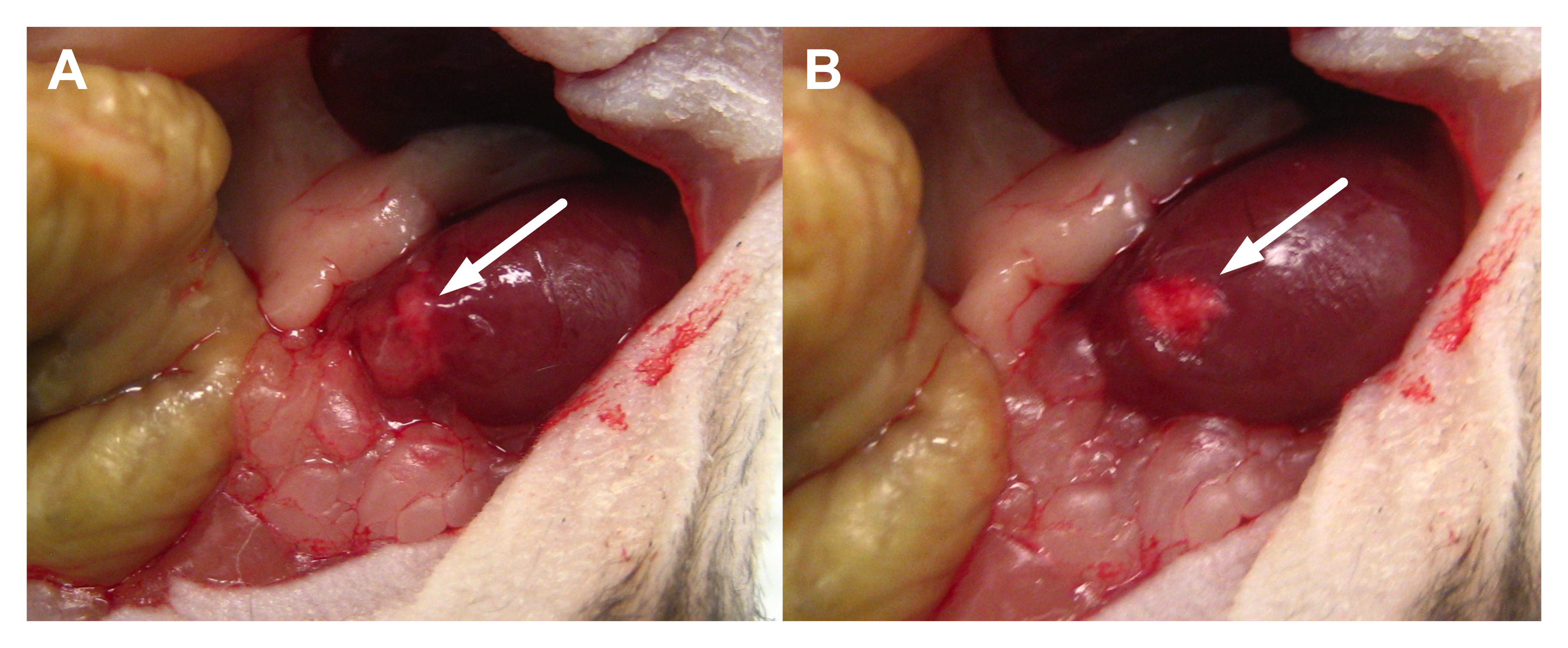

Supplement: Figure S1 — The surgical demonstration of the islet transplantation. (A) Transplanted islets in the islet group. (B) Transplanted islets in the islet/scaffold group. The white arrow indicates the transplantation site. (TIFF) [file pone.0057635.s001.tiff]
