# Supplementary material for: The Therapeutic Role of Monocyte Chemoattractant Protein-1 in a Renal Tissue Engineering Strategy for Diabetic Patients
Source: PLoS One. 2013 Feb 25;8(2):e57635. doi: 10.1371/journal.pone.0057635 (PMC3581514; doi:10.1371/journal.pone.0057635)
Supplement: Table S1 — Concentrations of plasma and urine proteins in non-diabetic C57BL/6 mice (age and sex matched). (DOCX) [file pone.0057635.s002.docx]

**Supporting Information**

Table S1 Concentrations of plasma and urine proteins in non-diabetic C57BL/6 mice

(age and sex matched)

|  | Week 0 (n=8) | Week 4 (n=8) | Week 8 (n=6) | Week 12 (n=7) |
| --- | --- | --- | --- | --- |
| Insulin (pg/ml) | 210.23±43.25 | 193.33±38.56 | 231.89±27.59 | 201.11±33.56 |
| Blood glucose (mg/dl) | 205.56±29.88 | 251.45±37.56 | 234.11±32.67 | 214.51±12.45 |
| Blood creatinine (mg/dl) | 0.78±0.11 | 0.69±0.09 | 0.72±0.23 | 0.84±0.15 |
| Urine creatinine (mg/dl) | 48.99±7.89 | 51.29±6.71 | 53.55±5.19 | 47.12±7.12 |
| Blood urea nitrogen (mg/dl) | 51.33±9.10 | 49.29±10.23 | 47.12±6.77 | 50.21±8.21 |
| Urine albumin (µg/24hr) | 10.27±1.34 | 17.56±4.23 | 13.45±5.37 | 20.11±6.77 |
